# The effect of positive thinking on resilience and life satisfaction of older adults: a randomized controlled trial

**DOI:** 10.1038/s41598-023-30684-y

**Published:** 2023-03-01

**Authors:** Zahra Taherkhani, Mohammad Hossein Kaveh, Arash Mani, Leila Ghahremani, Khadijeh Khademi

**Affiliations:** 1 Department of Health Promotion, School of Health, Shiraz University of Medical Sciences, Shiraz, Iran; 2 Department of Health Promotion, School of Health, Institute of Health, Research Center for Health Sciences, Shiraz University of Medical Sciences, Shiraz, 71536-755 Iran; 3 Department of Psychiatry, School of Medicine, Institute of Health, Research Center for Health Sciences, Shiraz University of Medical Sciences, Shiraz, Iran; 4 Student Research Committee, Department of Health Promotion, School of Health, Shiraz University of Medical Sciences, Shiraz, Iran

**Keywords:** Psychology, Health care

## Abstract

The cumulative effects of adversity and unhappiness affect life satisfaction and quality of life in the growing older adult population. Most of the interventions aimed at improving the health and quality of life of older adults have adopted a problem-oriented or weakness-focused approach. However, a positive or strengths-focused approach can also have a virtuous but more effective capacity to contribute to the well-being and life satisfaction of older adults. Therefore, the present study was conducted to investigate the effect of positive thinking training on improving resilience and life satisfaction among older adults. A randomized controlled trial was conducted on 100 older adults with simple random sampling. The intervention group received 90-min weekly sessions for eight weeks on positive thinking training through written homework for reflection, group discussion, and media. The data were collected using Ingram and Wisnicki Positive Thinking Questionnaire, Connor-Davidson Resilience Scale, and Tobin Life Satisfaction Questionnaire at baseline and one week and two months after the training. The collected data were analyzed using descriptive and inferential statistics in SPSS software 26. *P* values below 0.05 were considered statistically significant. Positive thinking training led to better thinking (*p* < 0.001), higher resilience (*p* < 0.001), and greater life satisfaction (*p* < 0.001). The study's findings showed the effectiveness of the positive thinking training approach in improving resilience and life satisfaction in older adults. It is recommended to evaluate the long-term outcome in populations with different social, economic, and cultural statuses in future studies.

## Introduction

The older adult population is growing because of improved healthcare and education systems. These individuals encounter physical, psychological, and social adjustments that challenge their sense of self and capacity to live. Many people experience loneliness and depression episodes in old age due to living alone or lacking a close family, which results in the inability to participate in community activities^1^. The simultaneous effects of health disorders experiences and socioeconomic problems throughout life lead to life satisfaction challenges and low tolerance of problems during old age^2^. Older adults care programs have often focused on secondary care for some physical and psychological illnesses with a negative health approach. Psychological research and theory on welfare and quality of life have also focused on the influence of negative aspects^3^. In contrast to this traditional attitude, however, the past two decades have witnessed the occurrence of an alternative positive viewpoint, which originates from developmental psychology and explores the personal and environmental factors that can improve psychological happiness and quality of life, particularly during hardship or stress^4,5^.

New positive approaches have focused on the importance and role of individual components and skills, including resiliency, and increased emphasis on ideal or successful aging^6^. Several features of resilience, including physical, mental, and social features, have been well-known among older people^7–9^, representing the multi-dimensionality of this feature. High resilience during later years of life has been accompanied by ideal outcomes, such as reduced depression and anxiety, increased quality of life, and improved lifestyle behaviors^10–12^. In general, individual factors, such as level of education, cognitive and emotional abilities, self-care skills, beliefs, and attitudes, as well as physical-social environment factors, including family, healthcare system, social welfare, and physical environment facilities for the safe presence of older adult's individuals in the community have vital effects on their physical and mental health. In this way, these factors can affect their efficiency and life satisfaction^13,14^. Studies have indicated that life satisfaction and happiness do not depend solely on external conditions; individual mental states, such as hope, self-esteem, and a sense of efficiency, are also important. Individuals with a positive mental structure can evaluate many negative events positively^15,16^. Resilience is one of the most important determining factors of older adults’ mental health. Resilience refers to individuals’ traits and skills that empower them to thrive in the face of hardship or a disruptive event^10,11^. Resilient people have flexibility, high confidence, life expectancy, the ability to forgive others, purposefulness, social participation, and a positive view of life and the future that prevents anxiety and depression in older adults^17^. In addition to internal and personal factors, external and environmental factors affect resiliency^18^. Thus, resilience is not a static phenomenon. In this context, resiliency can be improved by interventions, such as positive thinking techniques, reviewing past events, having social participation, strengthening self-confidence and motivation, using a semantic approach, e.g., yoga, and reinforcing internal powers^19,20^.

The pattern of individual thinking is important; individuals with positive thinking can overcome problems, while negative thoughts can lead to greater problems. Therefore, people can overcome life difficulties and events by abandoning negative thoughts and replacing them with positive ones^21,22^. The positive approach aims to identify the structures and methods that lead to well-being, happiness, and increased life satisfaction. Therefore, using positive training techniques to increase older adult's individuals’ resilience can act as a barrier to the physical and emotional weaknesses of older adults. Studies in Iran have evaluated the impact of positive thinking on older adults' mental health, psychological well-being, life expectancy, and loneliness^23–25^. However, these studies have not been able to directly investigate the impact of positive thinking on the improvement of resilience in this population. In other countries, the effects of positive thinking skills on increasing the resilience level have been somewhat proven^18,23,26^. However, resilience is an individual-social process that is significantly affected by culture and religion. Even the aging phenomenon is defined differently in various societies^19,24^.

In Iranian society, older adults are highly respected, and there is a positive attitude toward their knowledge and experiences, which gives them a deep sense of usefulness. However, negative stereotypes about their abilities prevent them from being active in society and interfere with their creativity, liveliness, and care priorities. Accordingly, overcoming these negative stereotypes leads to a sense of empowerment, resilience, and life satisfaction. Thus, providing effective educational, emotional, cultural, and social interventions can be helpful^27^.

Hence, the results of these studies cannot be generalized to Iranian society without independent evaluations. To our knowledge, no empirical studies were found in the current research framework. Additionally, the previous studies have only considered a part of the relationships, and a few similar studies have yielded inconclusive results. Considering the transition to old age in Iranian society and considering cultural, social, religious, and economic factors that vary among communities, the present study aims to investigate the effects of positive thinking training on increasing the resilience level and life satisfaction in a population of Iranian older adults.

## Methods

### Ethical considerations

This study was conducted with the clinical trials registration number IRCT20171212037844N1 and the registration date of 15/01/2018. In addition, all methods were carried out following relevant guidelines and regulations. The study objectives and procedures were explained, and the participants were asked to sign the written informed consent forms.

### Study design and population

**Figure 1:**
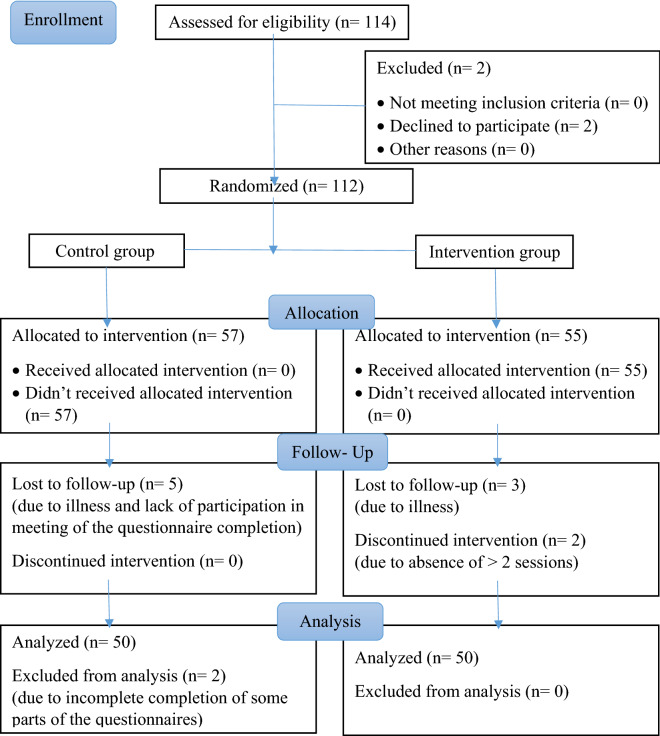
The CONSORT diagram of the study**.**

This educational randomized controlled trial was conducted in Shiraz, Iran, in 2018. The study population included people aged 60–70 covered by the older adult daycare centers affiliated with Welfare Organization in Shiraz. Based on a similar study^23^, using the appropriate formula with the type I error rate of 5% and the test power of 80%, considering a 10% attrition rate, the sample size was calculated as 45 participants in each group. Participants were selected in each center with the simple random sampling technique. For this purpose, the list of names of older adults covered by the center was considered the basis for sampling. The sampling interval was calculated by considering almost equal sex proportions using a randomization number between 1 and 10. The selected people were checked in terms of the inclusion criteria, and in case of ineligibility, the next person was selected from the list. All the participants had normal vision and hearing, scored ≥ 26 on the Mini-Mental State Examination (MMSE), and had the physical and mental ability to answer the questions. The participants had no psychiatric or neurological disorders and received no psychoactive drugs. Writing and reading literacy was another inclusion criterion. However, the exclusion criteria were being absent for more than two sessions, participating in similar training courses, and reluctance to continue participating in the study. The CONSORT diagram of the study is shown in Fig. 1.

### Data collection

The participants were asked to complete four questionnaires, including the Persian version of the Ingram and Wisnicki Positive Thinking Questionnaire^28^, the Connor-Davidson Resilience Scale^29^, the Tobin Life Satisfaction Questionnaire^30^, and a demographic information questionnaire.

The demographic data included age, sex, level of education, marital status, having children, housing situation, employment status, monthly income, and suffering from diseases.

Ingram and Wisnicki's Positive Thinking Questionnaire contained 30 items, e.g., *"I have a good sense of humor";* scored using a 5-point Likert-type scale ranging from 1 (never) to 5 (always). Thus, the total score of this questionnaire ranged from 30 to 150, with an average score of 90. Total scores above 90 and closer to 150 indicate a higher degree of positive thinking. The reliability index of this questionnaire was found to be 0.92 using Cronbach’s alpha^28^, which was 0.96 in the present study.

Connor-Davidson Resilience Scale included 25 items graded on a five-point Likert scale ranging from 1 (always false) to 5 (always true), with higher scores reflecting greater resilience. An example of the items in this questionnaire is *"I take pride in my achievements."* The reliability of the questionnaire was calculated with Cronbach’s alpha of 0.89^29^, which was 0.97 in the present study.

Tobin Life Satisfaction Questionnaire consisted of 13 items. Five items had negative contents, e.g*., "I have gotten more of the breaks in life than most of the people I know."* The rest had positive content, e.g.,* "I have made plans for things I’ll be doing a month or a year from now."* The statement *“I do not know”* was assigned two scores. In addition, positive statements received three scores for positive responses and one for negative ones. In contrast, negative statements were assigned three scores for negative responses and one for positive ones. Thus, the total life satisfaction score ranges from 13 to 39, with higher scores representing a higher level of life satisfaction. The reliability coefficient was calculated as 0.93. Cronbach’s alpha and Guttmann coefficients were found to be 0.79 and 0.78, respectively^30^. Cronbach’s alpha was 0.96 in the present study.

Baseline (Pre-test) and demographic data were collected one week before starting the training sessions. Post-intervention data were collected one week (Post-test) and two months (Follow-up) after the end of the intervention.

### Procedure and intervention

**Figure 2:**
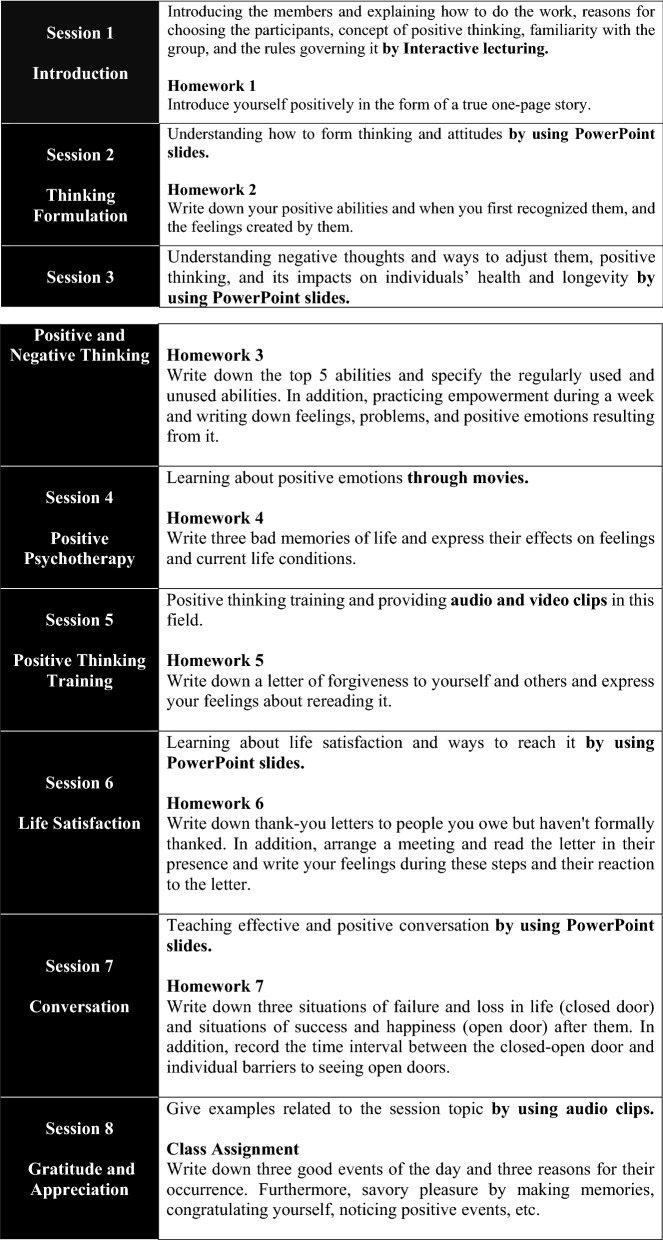
The topics and objectives, and homework of each training session.

The individuals in the intervention group participated in training sessions on positive thinking consisting of one 90-min session per week for eight consecutive weeks. The session's content was based on the theories of positive psychology^31,32^ in the field of positive thinking, including Strengths of Character (Homework 1, 2, 3), Engagement and Flow (Homework 4, 5, 6,7), and Meaning (Class Assignment). The training was performed using teaching/learning methods, including Interactive lecturing, group discussion, and media, such as PowerPoint presentations and audio and video clips. Based on scientific evidence, writing thoughts, feelings, and experiences, especially in combination with their expression, strengthens the character, reduces distress, and improves mental health. It is important to note that group discussions are more effective in these cases^33–37^. Hence, at the end of each session, participants were given written homework for reflection at home to practice positive thinking; they explained and discussed it in the group at the beginning of the next session (Except session 8). It should be noted that their practical experience was introduced as a method of positive thinking. The topics, as well as the objectives of each training session, are summarized in Fig. 2. In addition, a detailed description of the intervention program is shown in Appendix A.

### Statistical analysis

The collected data were analyzed using descriptive and inferential statistics in the SPSS software version 26^38^ and G-Power 3.1.9.2 (Düsseldorf, Germany). Data normality was checked by Shapiro–Wilk test. Additionally, a chi-square test was used to analyze the demographic data. Moreover, Repeated Measures ANOVA and independent T-test were performed to compare the research constructs' means. Bayesian inference was also used to update the probability of the hypothesis as more evidence or information became available. In all statistical tests, a *p *value below 0.05 was considered statistically significant.

### Ethics approval and consent to participate

All the participants were required to complete a written informed consent form. This study was approved by the Ethics Committee of Shiraz University of Medical Sciences (IR.SUMS.REC.1396.122).

## Results

The descriptive findings of this study showed no statistically significant differences in terms of the mean age (year) between the control group (M = 64.58, SD = 3.7) and the intervention group (M = 65.32, SD = 3.63); there was moderate evidence for the equal mean of age between two groups [t (98) = -0.639, *p* = 0.52; BF = 5.36]. The frequency distribution of other demographic data in the control and intervention groups is summarized in Table 1.

**Table 1:** Demographic characteristics of the participants in the study groups.

Characteristics	Groups				
	Control (n = 50)		Intervention (n = 50)		Chi-square test
	n	%	n	%	
Gender					*p* = 0.548
Male	25	50	28	53	
Female	25	50	22	47	
Education					*p* = 0.609
Illiterate and primary school	1	2	0	0	
Below high school	19	38	15	30	
High school graduate	17	34	23	46	
Bachelor’s degree	11	22	11	22	
Master’s degree or higher	2	4	1	2	
Marital status					*p* = 0.475
Married	29	58	24	48	
Single	2	4	6	12	
Divorce	3	6	3	6	
Widowed	16	32	17	34	
Child					*p* = 0.461
Yes	45	90	47	94	
No	5	10	3	6	
Housing situation					*p* = 0.212
Personal house with children	37	74	30	60	
Personal house alone	8	16	17	34	
Children’s house	3	6	2	4	
Others	2	4	1	2	
Job					*p* = 0.636
Unemployed	3	6	4	8	
Government employee	7	14	4	8	
Self-employed	3	6	2	4	
Retired	30	60	28	56	
Homemaker	7	14	12	24	
Income					*p* = 0.712
Less than one million Tomans	8	16	5	10	
One-two million Tomans	32	64	33	66	
Two–three million Tomans	8	16	8	16	
More than three million Tomans	2	4	4	8	
Diseases					*p* = 0.420
Yes	26	52	30	60	
No	24	48	20	40	
Type of disease					*p* = 0.869
Diabetes	11	22	12	24	
Cardiovascular disease	13	26	16	32	
Cancer	2	4	2	4	

Accordingly, there was no significant difference between the two study groups (*p* > 0.05). Most participants had high school and lower degrees, were married, had children, lived in their own houses, were retired, received one-two million Tomans as their income, and suffered from at least one underlying disease. The most and least frequent diseases were cardiovascular disorders and cancer, respectively.

The results revealed anecdotal evidence for the equal mean score of positive thinking between the control and intervention groups before the intervention (BF = 1.69). However, moderate evidence for the difference was observed between the two groups in this regard 1 week after the intervention [t (98) = −2.87, *p* = 0.005] and in the follow-up phase [t (98) = −2.73, *p* = 0.007]. In the intervention group, there was very strong evidence for the mean scores difference of positive thinking in the pre-test, post-test, and follow-up stages [F (1.05, 51.89) = 50.48, *p* < 0.001], and the effect of the intervention was maintained over time (Table 2).

**Table 2:** The mean scores of positive thinking in the study groups.

Positive Thinking	Baseline		1 week after intervention		8 weeks after intervention		F (df)	η^2^	Bayes Factor
Groups	Mean	SD	Mean	SD	Mean	SD			
Control (n = 50)	115.54	16.27	120.54	14.83	120.74	13.79	5.12 (1.19)	0.09	1.82
Intervention (n = 50)	110.04	16.1	128.86	14.1	127.96	12.54	50.48*(1.05)	0.5	0.03
t (df)	1.69 (98)		− 2.87 (98)		− 2.73 (98)				
*p*	0.09		0.005		0.007				
Cohen's d	0.33		0.57		0.54				
Bayes Factor	1.69		0.15		0.21				

*p < 0.001.

The results indicated moderate evidence for the equal mean of resiliency score at the pre-test stage between the control and intervention groups (BF = 5.69). Nonetheless, extreme evidence for the difference was found between the two groups in this regard after the intervention [t (87.5) = −11.152, *p* < 0.001] and in the follow-up phase [t (98) = −5.81, *p* < 0.001]. In the intervention group, there was extreme evidence for the difference in the mean score of resiliency in the pre-test, post-test, and follow-up stages [F (1.68, 82.61) = 77.16, *p* < 0.001] (Table 3).

**Table 3:** The mean scores of resilience in the study groups.

Resilience	Baseline		1 week after intervention		8 weeks after intervention		F (df)	η^2^	Bayes Factor
Groups	Mean	SD	Mean	SD	Mean	SD			
Control (n = 50)	79.08	13.7	80.34	14.33	80.62	12.93	2.87 (2)	0.07	5.45
Intervention (n = 50)	80.46	12.35	107.9	9.99	94.7	11.22	77.16*(1.68)	0.5	0.01
t (df)	− 0.59 (98)		− 11.152 (87.5)		− 5.81 (98)				
*p*	0.59		< 0.001		< 0.001				
Cohen's d	0.1		2.16		1.15				
Bayes Factor	5.69		< 0.001		< 0.001				

*p < 0.001.

Anecdotal evidence was found between the control and intervention groups regarding the equal mean of life satisfaction score in the pre-test stage (BF = 1.72). However, extreme evidence for the difference was found between the control and intervention groups in this regard after the intervention [t (98) = −4.53, *p* < 0.001] and in the follow-up phase [t (98) = −4.43, *p* < 0.001]. In the intervention group, there was strong evidence for the difference in the life satisfaction scores in the pre-test, post-test, and follow-up stages [F (1.23, 60.71) = 69.60, *p* < 0.001] (Table 4).

**Table 4:** The mean scores of life satisfaction in the study groups.

Life satisfaction	Baseline		1 week after intervention		8 weeks after intervention		F (df)	η^2^	Bayes Factor
Groups	Mean	SD	Mean	SD	Mean	SD			
Control (n = 50)	23.78	4.98	24.32	5.3	24.48	5.13	1.88 (1.43)	0.03	1.54
Intervention (n = 50)	22.24	4.08	28.98	4.95	28.9	4.83	69.60*(1.23)	0.58	0.09
t (df)	1.68 (94.40)		− 4.53 (98)		− 4.43 (98)				
*p*	0.09		< 0.001		< 0.001				
Cohen's d	0.35		0.9		0.88				
Bayes Factor	1.72		0.001		0.001				

*p < 0.001.

## Discussion

The main purpose of the present study was to investigate the effect of positive thinking training on increasing resilience levels and life satisfaction in a population of Iranian older adults. Designed positive thinking interventions are very sparse, specifically those targeting older adults. Most existing projects and studies have focused on high-risk younger adults, most of which are theoretical research studies with methodological weaknesses, small populations, and no exact outcome measures, which are inaccessible or invalid for older adults. Considering the cultural and social differences amongst societies, the lack of a similar study on an Iranian population, and low scores of positive thinking, resilience, and life satisfaction among Iranian older adults based on the present study findings, it seems necessary to perform appropriate interventions. The results of this study showed that the positive-thinking intervention significantly increased the mean scores of positive thinking, resilience, and life satisfaction in the intervention group one week and two months after the intervention. Several studies have been conducted on promoting resilience through older adults’ capability to enjoy positive experiences, activities to predict future events, and tools to reinforce relationships that activate feelings of pleasure and well-being^17^. Similarly, Ruiz-Rodríguez et al. conducted an interventional study and reported a significant relationship between social support, positive thinking, and social relationships with the degree of resilience^39^. Lysne PE et al. reported a significant positive correlation between resilience and different dimensions of health and life satisfaction^40^. Resiliency leads to life satisfaction through improving mental health and indirectly affects life satisfaction. In other words, resilience leads to a positive attitude and life satisfaction by affecting individual feelings and excitement^41^. The current study's clear and practical results can be implemented as training programs for many similar aging centers. However, this study had several limitations, including limited follow-up for only two months, selection of the study groups from older adult centers, and non-generalizability of some findings, such as resilience due to ethnic and cultural differences.

## Conclusions

In conclusion, the current study findings revealed a relationship between resilience and positive thinking. Positive thinking and interventions can increase older adults’ resilience, and thereby improve their quality of life. High quality of life can lead to greater life satisfaction. In addition, positive psychological training can directly contribute to positive and healthy thinking, ultimately leading to a better dynamic life for older adults.

## Supplementary Information

Supplementary Information.

## Data Availability

The datasets used and/or analyzed during the current study are available from the corresponding author upon reasonable request.

## Abbreviation

MMSE: Mini-Mental State Examination
