# Supplementary material for: The effect of positive thinking on resilience and life satisfaction of older adults: a randomized controlled trial
Source: Sci Rep. 2023 Mar 1;13:3478. doi: 10.1038/s41598-023-30684-y (PMC9977771; doi:10.1038/s41598-023-30684-y)
Supplement: Supplementary file 1 — Supplementary Information. [file 41598_2023_30684_MOESM1_ESM.docx]

**Appendix A**

**Contents of Educational Classes on the Intervention of Positive Thinking**

**First Session**

**Positive Introduction**

Please write a positive introduction of yourself in one page through a story so we could evaluate it next session and put it up for discussion. Your story should reflect on reality and represent you at your best. It should also have a beginning, middle and end with a strong ending. Please write your story comfortably or type it if you wish.

**Second Session**

The capabilities that I can identify in myself for my positive introduction are:

The first time I realized I have these capabilities were:

I have used these capabilities in the following fields and at the following incidents:

How did writing this positive introduction make you feel?

**Third Session:**

My top five positive capabilities:

1.

2.

3.

4.

5.

Which one of these capabilities is regularly practiced by you presently? Please explain how they benefit you.

Were there any capabilities which you benefited from in the past but no longer benefit from? If yes, please explain when and how you implemented these capabilities.

One way I can implement my …… capability in this week is:

In this week, find some space in your schedule to implement the mentioned capabilities. After it is done, provide short answers to the following questions:

1. How did implementing your capabilities make you feel?
2. Did you face any problems in the process? If yes, provide a short explanation on what these problems were and how you managed to overcome them.
3. Did you experience any positive emotions during and after finishing this schedule? If yes, describe what they were and when they appeared.

**Forth Session**

**Instructions on Writing Three Bad Memories**

Memories can be impactful. In fact, past memories usually influence our current state, whether they are good or bad. Please bring to mind three memories and write about them. We will discuss them in the next session.

**First Memory**

How does this memory influence your current state? Does this memory make you feel anger, sadness, or other symptoms of depression? Please write what comes to your mind after reading these questions.

**Second Memory:**

How does this memory influence your current state? Does this memory make you feel anger, sadness, or other symptoms of depression? Please write what comes to your mind after reading these questions.

**Third Memory:**

How does this memory influence your current state? Does this memory make you feel anger, sadness, or other symptoms of depression? Please write what comes to your mind after reading these questions.

**Fifth Session**

**Writing a Letter of Forgiveness:**

After writing this letter, please answer the following questions:

1. How did you feel as you were writing your forgiveness letter (or what was the feeling like)?
2. Did you feel like you were heart fully and actually willing to forgive? If yes, why? If no, why?
3. How did you feel after finishing and re-reading the letter?

**Sixth Session**

**Instructions on Writing a Letter of Appreciation (Gratitude)**

Expressing appreciation and gratitude is a beneficial practice for the improvement of satisfaction and increasing positive emotions about the past. Although most people usually say “Thank you”, they don’t heartfully and genuinely mean to appreciate those who help them in some way or another. Think of some people in your life whom you are indebted to, but you never officially or properly expressed gratitude towards them. Write a letter of appreciation for such people and provide details.

Please note that the letter should clearly mention what this individual has done for you and how it has impacted your life. If necessary, rewrite this letter and when you decided that it is well-written, schedule a meeting and personally give the letter to him/her. Don’t explain too much about the purpose of your meeting. In your meeting, read the letter to him/her or let him/her read it in your presence. After you finished writing or presented your letter of appreciation, ponder for a few minutes and answer the following questions:

1. How did you feel when writing the letter?
2. How did you feel when you were getting ready to present your letter? Was it hard or easy to do?
3. How did the person whom you wrote the letter to react? How did his/her reaction influence you?
4. If expressing gratitude in this way has led to the emergence of positive emotions in you, please state how long it lasted after presenting the letter? Does remembering that moment and its emotions impact your current emotions and mental state? Please explain.

**Seventh Session**

**Instructions on the Opened and Closed Doors Homework**

What allows optimists to be happier than pessimists in that they tend to focus on the positive aspects of matters. Optimists do not only think positively, rather they focus on the positive aspects that really exist. The old expression “to see the glass half full or half empty” illustrates that optimists focus on the positive aspects and the full half of the glass which is a reality, and the pessimists focus on the negative side of reality which is the empty half. Optimists are not naïve, crude, or stupid.

This practice has been designed to help you think of those times in your life when important doors were closed in your pathway, but then certain doors were also opened (the expression “when one door closes, another opens”).

Please write about three times in your life when you lost something important like when what you had planned failed or when someone discarded you. Then, think of the doors which opened in your path after these failures or losses.

1. The door that closed:

And the one that opened afterwards:

1. The door that closed:

And the one that opened afterwards:

1. The door that closed:

And the one that opened afterwards:

Ponder for a moment and provide short answers to the following questions.

1. How long after these doors closed, could you see the ones that opened up?
2. If something stops you from seeing the opened doors, what do you think it is?
3. In the future when certain doors close in your path, what can you do to see the opened doors sooner?

**Eighth Session**

**Instructions on Joy Tasting**

One of the ways to increasing positive emotions is to taste the joys of life. We want you to plan at least one enjoyable activity and carry it out by implementing the methods of joy tasting. This concept consists of the awareness of joy and paying purposeful attention to the experience of it.

To taste the joy of an activity or phenomenon these methods can be used:

1. Discussing the joyful experience with others

Before carrying it out, share the experience with others and if possible, make them join you. Later on, discuss the experience with other and talk about it.

1. Making memories

Take notes in your mind of the phenomenon as it happened and later review the memory. If you like you can also take pictures or buy some souvenirs that make you remember the positive experience.

1. Congratulate yourself

Congratulate yourself for what happened and be proud of yourself.

1. Be aware

Pay attention to the details and elements of the positive experience and stop the thoughts that distract you and destroy your pleasure (Pleasure killing thoughts disrupt the Joy tasting experience. This happens at three times: when you think that your positive experience is not as good as others, when you think that you should have done what you could or should have instead of what you did, and when irrelevant thoughts distract you from your joyful experience).

1. Attraction

Let the phenomena completely attract your attention and drown you in itself.

Please write down your plan for the tasting of the joy of a positive experience.

If possible, schedule joyful activities for half of the day or as we suggested, for the full day and start carrying them out. You must immerse these activities with you schedule and resist against seizing them and doing something else.

Joy tasting activities that I planned to do on …… at ……… are:

Joy tasting methods that I will implement for this activity are:

Please answer the following questions after finishing your joy tasting activities:

1. Which method did you use for tasting joy and which one was more suitable for you?
2. Did you experience any pleasure killing thoughts? Could you resist against them? If yes, explain how you did so.

**Instructions on Giving Thanks by Writing**

For most of us it is easier to think about the bad things that happen throughout the day, rather than the good incidents that happen. This inclination towards negative matters and ignorance towards the positive, is a type of biased function in the way human beings tend to think which is called fundamental negativity bias.

In the coming week, we want you to write three good things that happen to you each day in a notebook so that you can get used to overcoming this fundamental negativity bias.

In addition to the daily writing of these three, we also want you to think about why they happened. For example, did you cause them to happen? Or did someone else cause this good incident to happen? Or was it completely accidental?

Pondering the good things that happen in our life help us to feel more positive emotions and have more satisfaction while developing certain capabilities such as optimism and giving thanks.

Date:

Three Good Events: Why they happened?

1. 1.

2. 2.

3. 3.

Date:

Three Good Events: Why they happened?

1. 1.

2. 2.

3. 3.

Date:

Three Good Events: Why they happened?

1. 1.

2. 2.

3. 3.
